# The Ergogenic Effects of Intermittent Palm Cooling on Repeated Baseball Throwing Are Reversed when Cooling-Induced Pain Occurs

**DOI:** 10.5114/jhk/194455

**Published:** 2025-05-29

**Authors:** Kun Han Lin, Yi Ming Huang, Zong Yan Cai

**Affiliations:** 1 Physical Education Office, National Tsing Hua University, HsinChu City, Taiwan (R.O.C.).; 2 Fascia Plus Lab, Taipei City, Taiwan (R.O.C.).; 3 Center for Physical and Health Education, Si Wan College, National Sun Yat-sen University, Kaohsiung City, Taiwan (R.O.C.).

**Keywords:** ergogenic aid, explosive strength, throw velocity, throw accuracy, arousal level

## Abstract

Intermittent palm cooling (PC) could benefit strength performance. However, its effect on baseball throwing, which requires explosive strength, has yet to be determined. This study aimed to examine whether intermittent PC would enhance repeated baseball throwing performance and how pain after PC would affect outcomes. Twenty-two university division II male baseball athletes were instructed to perform five sets of 10 throws at maximum speed toward a target, separated by 3-min of recovery with PC into 10°C water or non-cooling (NC). Participants were classified into the no-pain group (NPG, n = 10) and mild-to-moderate pain group (PG, n = 12) according to their pain sensation after PC for data analysis. The results showed a significantly lower rating of perceived exertion under PC conditions (p < 0.05). In the NPG, PC resulted in higher mean throw velocity (107.9 ± 9.9 vs. 106.9 ± 10.2 km/h), maximal throw velocity (112.5 ± 9.0 vs. 111.2 ± 9.8 km/h), accuracy, and the arousal level than NC; in the PG, PC resulted in lower mean throw velocity (101.0 ± 11.1 vs. 105.2 ± 10.5 km/h) and maximal throw velocity (105.0 ± 10.8 vs. 108.7 ± 9.8 km/h) when compared with NC (p < 0.05). However, no significant difference was noted in accuracy and arousal levels between PC and NC (p > 0.05) in the PG. In conclusion, after PC, pain-free baseball athletes can enhance their baseball throwing performance. The presence or absence of pain after PC can serve as an initial assessment of whether to use it as an ergogenic aid strategy.

## Introduction

Throwing performance, including velocity and accuracy, is crucial in baseball (Freeston and Rooney, 2014). During routine training or competition, baseball athletes repeatedly exert maximal throwing efforts with brief recovery periods. Hence, it is crucial to explore potential ergogenic aids that can assist baseball players in achieving excellent repeated throwing performance and accelerating recovery between rest intervals. This is essential for preventing performance decrements and succeeding in competition.

During baseball training or competition, athletes commonly apply ice packs directly to their shoulders or arms during rest intervals to alleviate discomfort from high-intensity exercise. Gradually, cooling strategies are frequently used as ergogenic aids in baseball games. In a study, Verducci (2001) applied 3 min of cooling treatments using ice packs filled with ice cubes to the shoulder and the forearm of six pitchers between four innings of 22 pitches per inning. The cooling strategy increased mean pitching velocity, whereas no differences were noted in accuracy. In a repeated-measures crossover design study, Bishop et al. (2016) instructed eight pitchers to pitch five innings including 12 pitches per inning in a simulated baseball game, separated by 6 min of the recovery period with local cooling on the deltoid muscle and the forearm of the pitching arm for 4 min or without cooling. That study found that intermittent local cooling attenuated velocity loss in baseball pitching and lowered the rating of perceived exertion (RPE) (Bishop et al, 2016). Previous research suggests that pain relief and diminished tissue temperature from intermittent local cooling may account for the attenuated velocity loss and facilitated recovery during repeated pitches (Bishop et al., 2016; Verducci, 2001). However, Huang et al. (2021) revealed that cooling interventions during real games may be limited because most pitchers prefer to keep their pitching arms warm rather than cold between innings. Furthermore, during short-term, explosive exercise, it is important to maintain the working muscles within the optimal temperature range (Olberding and Deban, 2017). If the muscles cool significantly, both concentric and eccentric isometric strength, as well as explosive strength, decreases (Cross et al., 1996; Ruiz et al., 1993). This may be due to vasoconstriction and reduced calcium release from the sarcoplasmic reticulum, impairing crossbridge formation (Ruiz et al., 1993). Additionally, it may impede the sensitivity of the neuronal discharge and diminish the sensitivity of muscle spindles, which in turn affects muscle contractile function (Ruiz et al., 1993). Therefore, direct cooling working muscles may not be beneficial for explosive movements.

Cooling working muscles directly can reduce performance, and hence, some have examined the benefits of cooling peripheral extremities, such as palms (Caruso et al., 2015; Grahn et al., 2012; Kwon et al., 2010) and feet (Cai et al., 2021), instead of the muscles themselves to avoid performance reduction due to decreased muscle temperature (Cross et al., 1996; Ruiz et al., 1993). These areas have abundant arteriovenous anastomoses and are sensitive to cold (Walløe, 2016). Therefore, peripheral cooling may maintain the facilitation of A-alpha motor neurons mediated by the activation of the cutaneous cold receptors (Manuel and Zytnicki, 2011), potentially causing a higher afferent stimulus (De Keyser et al., 2018). Although the mechanism of peripheral cooling in increasing strength performance is unclear, it seems to elicit a higher arousal level (Hopkins et al., 2002; Palmieri-Smith et al., 2007; Pietrosimone and Ingersoll, 2009; Wu et al., 2021, 2023), increase motor unit recruitment (Cai et al., 2021; Kwon et al., 2010; Wu et al., 2021), and delay the onset of fatigue (Caruso et al., 2015; Kwon et al., 2010; Wu et al., 2023), which may be the reason for its effectiveness.

During exercise, palm cooling (PC) is a more convenient and easily implemented method. Previous studies have determined that intermittent PC increased the number of repetitions and total exercise volume during a 4-set 85% of 1-repetition maximum (1RM) bench press workout accompanied with a reduced RPE (Kwon et al., 2010), increased the number of repetitions to failure in pull-up exercise (Grahn et al., 2012), and delayed the average power decrements in a 4-set 8-repetition of concentric flywheel leg press resistance exercise at a submaximal level of effort with hastened blood lactate clearance (Caruso et al., 2015). These findings indicate that intermittent PC has a beneficial effect on strength performance. Baseball throwing is an exercise that requires explosive strength. However, the effect of PC on baseball throwing speed is uncertain.

Furthermore, previous studies examining the impact of PC on exercise performance have excluded participants who did not experience or report cooling-induced pain sensation (Caruso et al., 2015; Grahn et al., 2012; Kwon et al., 2010). Pain can induce changes in motor performance, motor unit recruitment, and rate coding that varies across different contraction speeds (Martinez-Valdes et al., 2021). Given that cooling-induced pain varies individually (Soeda et al., 2021), and cooling reduces force production in the presence of pain (Chi et al., 2012; Roatta and Farina, 2011), it is important to evaluate the effect of PC on explosive power performance categorized by cooling-induced pain. Moreover, considering that hand cooling has been shown to decrease fingertip dexterity and hand gross movement (Cheung et al., 2008, 2023) and muscle contraction due to shivering disrupts fine motor control (Wakabayashi et al., 2015), the effect of PC on throwing accuracy should be further investigated.

Within this framework, this study aimed to investigate whether the application of intermittent PC would have an ergogenic effect on throwing velocity and examine whether PC would affect throwing accuracy. Perceptual responses during the workout were measured as a secondary outcome. The results were divided into two groups based on whether the participants experienced pain from cooling. We hypothesized that PC would increase baseball throwing velocity, but decrease throw accuracy in participants who did not feel cooling-induced pain and decrease baseball throwing velocity and throw accuracy in participants who felt cooling-induced pain.

## Methods

### Participants

The study included 22 university division II baseball players with the following characteristics: age (mean ± standard deviation [SD]) 21.4 ± 1.4 years, body height 172.5 ± 4.6 cm, body mass 69.7 ± 8.7 kg, and baseball training experience 7.8 ± 3.5 years. The eligibility criteria were as follows: male baseball players aged ≥20 years who had been performing regular baseball training (>3 times/week) for at least three years and were classified as healthy with no major medical history. Conversely, the exclusion criteria were as follows: a history of injury in the previous 6 months and taking ergogenic supplements or medicine that could affect throwing performance.

Participants were instructed to maintain their regular dietary patterns during the study. They were also required to abstain from caffeine and alcohol for 24 hours before each testing session and to avoid strenuous exercise throughout the study period. The recruited participants were fully informed about the experimental procedure, potential risks, and benefits of the study, and they provided informed consent. The study was conducted according to the guidelines of the Declaration of Helsinki and approved by the National Cheng Kung University Human Research Ethics Committee (approval code: NCKU HREC-E-112-610-2; approval date: 04 December 2023).

### Study Design

This study was conducted for 3 days during the off-season period, including 1 day of familiarization followed by 2 days of experimental sessions. A randomized, counterbalanced, within-group research design was used to compare the effects of PC and test our hypotheses. During each experimental session, participants were instructed to aim at the target as accurately as possible and throw with maximal speed. The target was 18.75 m away (distance between the pitcher’s mound and the home plate). The throwing protocol consisted of five sets (3-min interval) of 10 fastballs, totaling 50 throws, under NC and inter-set PC conditions, within a 5-d interval.

**Figure 1:**
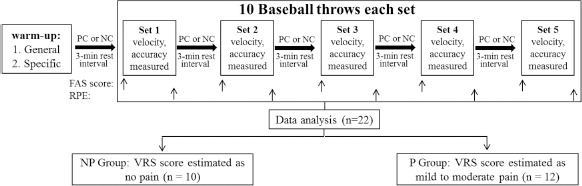
Schematic diagram of the experimental testing protocol and the flow chart of data analysis. FAS = felt arousal scale; RPE = rating of perceived exertion; VRS = verbal rating scale

To further explore the potential effect of PC-induced pain on experimental results, participants experiencing varying levels of pain during the PC process were categorized into the no pain group (NPG) and the mild-to-moderate pain group (PG) according to the 4-point verbal rating scale (VRS) score (Relvas et al., 2016) measured after the PC condition for statistical analysis (Figure 1).

### Study Procedures

Initially, participants were familiarized with the study procedures, and individual anthropometric data were collected. Standard instructions for the scale procedures were also provided to ensure accurate perceptual responses. Experimental sessions started with a standard general warm-up, as shown in Figure 1, including low-to-moderate-intensity jogging and dynamic/static stretching for each major muscle group. A sport-specific warm-up including gradually increasing the intensity of tossing and catching the baseball was then conducted. Participants threw 12 baseballs at a distance of 10 m at ~50% (as perceived by themselves) of the their fastest throwing speed, followed by throwing 12 baseballs at a distance of 20 m at ~70% of the their fastest throwing speed, and then 12 baseballs at a distance of 30 m at ~90% of the their fastest throwing speed. After the warm-up, baseball players performed five sets of 10 maximal effort fastball throws (15-s interval), separated by 3 min of recovery with or without inter-set PC by immersing both hands in 10°C cold water for 2.5 min. Before each set of throwing under the PC condition (after cold-water immersion), participants' perceived arousal levels were assessed using the Felt Arousal Scale (FAS). Immediately after each set of throwing, participants were requested to rate their RPE of that set using the Borg CR-10 scale. All experimental sessions were performed at the same time of day (6:00–7:30 pm) in an outdoor baseball field. The temperature remained stable throughout the experimental periods, ranging from 24°C to 26°C. All experimental sessions were conducted under the strict and direct supervision of experienced researchers with expertise in baseball.

### Measures

#### Palm Cooling Intervention

Baseball players immersed their palms up to the wrist in buckets of water (Streff et al., 2010) at a controlled temperature of 10°C ± 1°C for 2.5 min. The temperature and cooling time were chosen based on previous studies that had shown the benefits of cooling extremities for strength performance (Cai et al., 2021; Kwon et al., 2010; Wu et al., 2021). The water temperature during the PC condition was monitored, and the target temperature was maintained by adding crushed ice when necessary (Cai et al., 2021; Wu et al., 2021, 2023). Immediately after each cold-water immersion, participants dried their palms.

#### Speed Measurement

A Stalker Radar Speed Gun PRO (Bushnell Outdoor Products, Overland Park, KS, USA) was used to measure baseball speed. The mean throwing velocity and maximal throwing velocity of participants during each session were chosen for statistical analysis. In the present study, mean throwing velocity was defined as the average value during 50 throws, whereas maximal throwing velocity was described as the highest speed obtained during each 50-throwing session.

#### Accuracy Measurement

The target was a 70-cm long and 60-cm wide mark, securely attached to a mesh net positioned 60–130 cm above the ground and 18.75 m from the participant's throwing position. The balls that hit the target were scored. The accuracy was assessed by calculating the hit rate of the target out of 50 throws (number of successful throws/50 throws × 100%).

#### FAS

The FAS has been used in previous exercise studies to gauge perceived arousal and has been demonstrated to have strong convergent validity (Van Landuyt et al., 2000). It is a 6-point scale, ranging from 1 to 6 points, with high scores indicating states such as excitement and low scores indicating states of relaxation (Van Landuyt et al., 2000).

#### RPE

Participants were requested to rate their perceived exertion using the Borg CR-10 scale.

#### VRS

Participants were instructed to assess their current pain level at the end of the PC condition using a 4-point VRS ranging from no pain to severe pain. The scale was defined as follows (Relvas et al., 2016): 0—no pain or discomfort; 1—mild pain: feeling pain, but no oral medication required; 2—moderate pain: feeling pain, but no oral medication required; 3—severe pain: feeling pain and is no longer able to perform any type of activity, feeling the need to lie down and rest.

### Statistical Analysis

Data were expressed as mean ± SD. A paired t-test was used to quantify the differences in throwing velocity and accuracy. A two-way repeated-measures analysis of variance (ANOVA) was conducted to assess differences in the FAS score and RPE values. Significant condition × set interactions were further analyzed with simple main-effects analyses. When no significant interaction was found, the least significant difference (LSD) post hoc tests were used to examine significant main effects for each condition and set. Cohen’s d was used as an effect size (ES) measurement for comparing two means, with interpretations of small (0.2), medium (0.5), and large (0.8) (Cohen, 1998). Statistical significance was set at p < 0.05.

## Results

After classifying by pain, this study included 10 NPG (point 0, n =10, no pain) and 12 PG (point 1, n = 6, mild pain; point 2, n = 6, moderate pain; point 3, n = 0, severe pain) adult participants. The demographic data are presented in Table 1.

**Table 1:** Descriptive data for the athletes, divided into the no pain group (NPG) and the pain group (PG).

Group	NPG (n = 10) Point 0 (n = 10)	Point 1 (n = 6)	PG (n = 12) Point 2 (n = 6)	Point 3 (n = 0)
Age (years)	20.8 ± 1.0		21.9 ± 1.6	
Body height (cm)	173.3 ± 5.4		171.8 ± 3.8	
Body mass (kg)	69.3 ± 7.0		70.0 ± 10.1	
Baseball training experience (years)	6.7 ± 3.9		8.7 ± 3.1	

Participants experiencing varying levels of pain during the PC process were categorized into the NPG and the PG according to the 4-point verbal rating scale score; 0 = no pain or discomfort; point 1 = mild pain; point 2 = moderate pain; point 3 = severe pain

**Figure 2:**
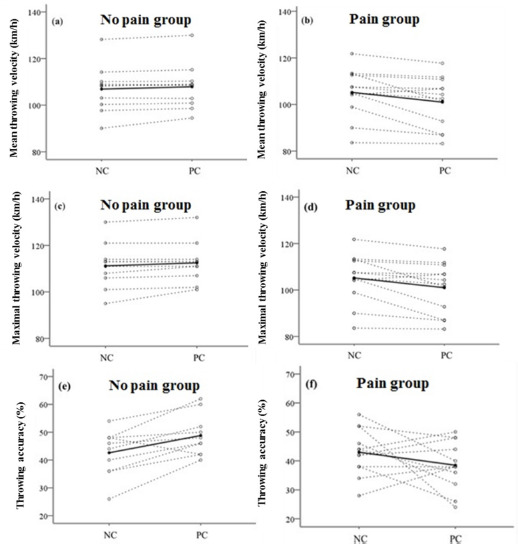
Individual (dotted lines, open circles) and average (solid line, filled circle) throwing performance under NC and PC conditions with the individual values. *(a) Mean throwing velocity in participants classified as experiencing no pain (n = 10) by the verbal rating scale (VRS) after the PC condition; (b) Mean throwing velocity in participants classified as having mild or moderate pain (n = 12) by the VRS after the PC condition; (c) Maximal throwing velocity in participants classified as experiencing no pain (n = 10) by the VRS after the PC condition. (d) Maximal throwing velocity in participants classified as having mild or moderate pain (n = 12) by the VRS after the PC condition; (e) Mean throwing accuracy in participants classified as experiencing no pain (n = 10) by the VRS after the PC condition; (f) Mean throwing accuracy in the participants who were classified as having mild or moderate pain (n = 12) by the VRS after the PC condition*

Figure 2 shows that for the NPG, PC treatments resulted in significantly higher mean throw velocity (107.9 ± 9.9 vs. 106.9 ± 10.2 km/h, *t* = 2.071, *p* = 0.034, *ES* = 0.323), maximal throw velocity (112.5 ± 9.0 vs. 111.2 ± 9.8 km/h, *t* = 2.112, *p* = 0.032, *ES* = 0.331), and throw accuracy (48.8% ± 7.1% vs. 42.6% ± 7.7%, *t* = 3.615, *p* = 0.003, *ES* = 0.566) compared with the NC condition. However, for the PG, PC treatments resulted in significantly lower mean throw velocity (101.0 ± 11.1 vs. 105.2 ± 10.5 km/h, *t* = −2.959, *p* = 0.007, *ES =* 0.443) and maximal throw velocity (105.0 ± 10.8 vs. 108.7 ± 9.8 km/h, *t* = −2.836, *p* = 0.008, *ES* = 0.422) when compared with the NC condition, and no significant difference in throw accuracy between the PC and NC conditions (38.5% ± 2.2% vs. 43.0% ± 2.1%, *t* = −1.497, *p* = 0.08, *E*S = 0.157).

Two-way ANOVA and post-hoc analysis of FAS and RPE data (Table 2) revealed that the NPG exhibited significantly higher FAS under the PC than the NC condition (*F* = 7.484, *p* = 0.23, *ES* = 0.454). However, for the PG, there was no significant main effect of condition on the FAS score (*p* = 0.91). A significant main effect was only observed in the set (*F* = 7.075, *p* < 0.001, *ES* = 0.391; Table 2).

**Table 2:** Felt Arousal Scale score and the rating of perceived exertion of the five sets of throwing

Condition	Set 1	Set 2	Set 3	Set 4	Set 5	mean	ANOVA (*p* values)	
**Felt Arousal Scale score in the no pain group (n =10)**								
Non cooling	3.2 ± 1.1	3.2 ± 1.0	3.3 ± 1.1	3.4 ± 0.8	3.4 ± 0.8	3.3 ± 0.8*	**Condition**	0.023
Palm cooling	4.2 ± 0.9	4.0 ± 0.8	4.0 ± 1.2	4.3 ± 0.9	3.9 ± 0.9	4.1 ± 0.7	**Set**	0.888
Pooled data	3.7 ± 1.1	3.6 ± 1.0	3.7 ± 1.1	3.9 ± 1.0	3.7 ± 0.9		**Interation**	0.584
**Felt Arousal Scale score in the pain group (n =12)**								
Non cooling	3.1 ± 1.2	3.4 ±1.0	3.7 ± 1.2	3.7 ± 1.2	3.7 ± 1.2	3.5 ± 0.9	**Condition**	0.091
Palm cooling	2.4 ± 0.9	2.8 ± 0.5	3.3 ± 0.9	3.4 ± 1.0	3.6 ± 0.7	3.1 ± 0.6	**Set**	< 0.001
Pooled data	2.8 ± 1.1	3.1 ± 0.8^a^	3.5 ± 1.0^a^	3.5 ± 1.0^a^	3.6 ± 0.9^ab^		**Interation**	0.559
**Rating of perceived exertion in the no pain group (n =10)**								
Non cooling	4.4 ± 1.3	4.9 ± 1.2	5.4 ±1.3	5.7 ±1.2	6.3 ±1.3	5.3 ± 1.1*	**Condition**	0.001
Palm cooling	3.3 ± 1.2	3.6 ± 0.8	4.4 ± 0.8	4.8 ± 0.8	5.1 ± 0.8	4.2 ± 0.8	**Set**	< 0.001
Pooled data	3.9 ± 1.3	4.3 ± 1.2	4.9 ± 1.1^ab^	5.3 ± 1.0^abc^	5.7 ± 1.2^abcd^		**Interation**	0.787
**Rating of perceived exertion in the pain group (n =12)**								
Non cooling	4.7 ± 0.7	5.0 ± 0.6	5.5 ± 0.7	6.1 ±1.0	6.7 ± 0.9	5.6 ± 0.6*	**Condition**	< 0.001
Palm cooling	3.5 ± 0.5	3.9 ± 1.1	4.7 ± 1.1	4.9 ± 1.3	5.5 ± 1.3	4.5 ± 1.0	**Set**	< 0.001
Pooled data	4.1 ± 0.8	4.5 ± 1.0^a^	5.1 ± 1.0^ab^	5.5 ± 1.3^abc^	6.1 ± 1.2^abcd^		**Interation**	0.671

* significant difference between NC and PC (p < 0.05),

a significant difference compared to the first set (p < 0.05),

b significant difference compared to the second set (p < 0.05),

c significant difference compared to the third set (p < 0.05),

d significant difference compared to the fourth set (p < 0.05)

For RPE data, comparisons between PC and NC conditions showed significantly lower RPE values in both the NPG (*F* = 26.561, *p* = 0.001, *ES* = 0.747) and the PG (*F* = 33.702, *p* < 0.001, *ES* = 0.754). Additionally, RPE values increased significantly during the throwing workouts in both the NPG (main effect of set, *F* = 31.171, *p* < 0.001, *ES* = 0.776; Table 2) and the PG (main effect of set, *F* = 31.138, *p* < 0.001, *ES* = 0.739; Table 2).

## Discussion

The present study used intermittent PC at 10°C for 2.5 min as an ergogenic cooling strategy. The strategy of peripheral cooling has been demonstrated to improve muscle strength performance (Cai et al., 2021; Kwon et al., 2010; Wu et al., 2021). In contrast, in this study, a cooling temperature of 10°C seemed to reach the 15°C cooling-induced pain threshold (Barati et al., 2017). Individual differences in cooling-induced pain could affect strength performance (Chi et al., 2012; Roatta and Farina, 2011), thus participants were divided into a NPG (n = 10) and a light-to-moderate PG (n = 12) for analysis. To the best of our knowledge, this study is the first to investigate the effect of various levels of PC-induced pain on baseball throwing performance. The primary findings of this study mostly confirmed our research hypothesis, indicating that intermittent PC increased arousal levels and enhanced both the mean throwing velocity and maximal throwing velocity in skilled baseball athletes who did not experience pain following intermittent PC treatment. However, intermittent PC decreased the mean throwing velocity and maximal throwing velocity in trained baseball athletes who experienced pain after intermittent PC treatment. Interestingly, intermittent PC increased throwing accuracy in trained baseball athletes who did not experience pain.

In the present study, the NPG participants exhibited higher arousal levels under the PC than the NC condition. Although the present study did not delve into the precise mechanisms of how arousal would affect strength, previous research has indicated that elevated arousal levels can enhance motor cortex excitability (Hajcak et al., 2007). Additionally, Schmidt et al. (2009) conducted a functional magnetic resonance imaging study to investigate the effect of arousal on motor command and force production. Their findings demonstrated that the arousal-related brain regions contributed to motor command and facilitated force production. Those researchers suggested that heightened arousal levels could benefit sports requiring maximal power, where performance could thus be enhanced in highly arousing situations. The present study found that cooling the palm during repeated baseball throwing had a similar effect on arousal levels to cooling the feet in a previous study (Wu et al., 2021, 2023). It has been observed that elevated arousal by foot cooling plays an ergogenic effect on lower limb strength performance (Wu et al., 2021). Conversely, PC yielded an ergogenic effect on strength performance in high-intensity bench press workouts and flywheel leg press exercises (Caruso et al., 2015; Kwon et al., 2010). Extending from previous studies that have found that PC is beneficial to strength performance, the present study further demonstrates that PC enhances throwing velocity, including mean and maximal throwing velocities, which are indicators of explosive strength performance. The intermittent PC increased both maximal and mean throwing velocity, indicating enhanced peak power performance and anti-fatigue by maintaining a higher average baseball throwing velocity during repeated throwing exercises. Moreover, unexpectedly, PC increased throwing accuracy, contrary to previous findings that hand cooling at 10°C for 5 min significantly decreased fingertip dexterity and hand gross movement (Cheung et al., 2023) and affected the accuracy of shoulder flexion performance due to cold shivering (Meigal et al., 1998). Methodological differences, such as cooling sites (palm vs. hands and forearms) and duration (immersion for 2.5 min vs. 5 min) may contribute to the observed discrepancy. Our study findings suggest that intermittent brief PC may not induce hypothermia or trembling in participants who do not experience cooling-induced pain, and therefore does not affect exercises requiring accuracy. The data in this study provided limited evidence to explain the exact mechanism of how PC increases throwing accuracy. Given that the optimal arousal level benefits sports requiring accuracy (Doyle et al., 2016), it is plausible that the moderately increased arousal levels in the present study may provide some rationale. In addition, compared to a previous study on baseball throwing using intermittent cooling of arms and shoulders without increasing accuracy (Verducci, 2001), the present study suggests that applying PC is a more effective strategy for improving the accuracy of baseball throwing.

In contrast, an opposite outcome was noted in throwing performance between PG and NPG players, exhibiting a decrease in both maximal throwing velocity and mean throwing velocity. The decrease in force production due to cooling is often accompanied by cooling-induced pain (Chi et al., 2012; Roatta and Farina, 2011) and a reduction in cutaneous sensory feedback (Caminita et al., 2020), which depends on cooling temperature and duration. As demonstrated by Roatta et al. (2011), pain caused by cooling decreases electrically stimulated muscle force. Another similar investigation observed that strength was reduced in the presence of pain (Chi et al., 2012). Caminita et al. (2020) also reported that reducing cutaneous sensory feedback by peripheral cooling at foot soles at 0°C for 15 min decreased squat jump height and vertical ground reaction force. Although pain data were not available in the study, previous investigations using the same cooling temperature and duration have shown that cooling-induced pain typically occurs (Chi et al., 2012; Roatta and Farina, 2011). This pain may decrease explosive performance by interfering with afferent stimulus to cutaneous cold receptors, resulting in reduced activation of A-alpha motor neurons. Regarding throwing accuracy, intermittent PC did not significantly affect the PG participants, supporting the idea that increased arousal levels may be partly responsible for the improved accuracy in the NPG. However, the present study found that half of the participants (Figure 2f) decreased throwing accuracy under the PC condition. Therefore, more attention should be paid when practically applying PC to training.

Various intermittent cooling strategies have been demonstrated to reduce the RPE during exercise, such as resistance exercise (Kwon et al., 2010), high-intensity exercise (De Koning et al., 2011), and baseball throwing (Bishop et al., 2016; Verducci, 2001). Previous research has shown that perception of effort is a key element in the development of fatigue, and improvements or impairment of performance are partly mediated by it (Pattyn et al., 2018). Performance improvements during repeated exercises are often accompanied by decreases in the RPE (Kwon et al., 2010; Verducci, 2001), and high-intensity exercise is partly regulated by the RPE (De Koning et al., 2011). Intermittent cooling strategies made participants feel like they were exerting less effort during exercise, and facilitated recovery, potentially leading to improved performance (Bongers et al., 2017; Verducci, 2001). In the present study, intermittent PC significantly decreased RPE values during the five sets of baseball throwing for all participants; however, those who experienced cooling-induced pain did not see performance improvement. In the present study, data classified by pain after PC reveal that cooling-induced pain may counteract the benefits of reduced perceived effort on improvements in athletic performance.

This study has some limitations. First, participants in the current study had undergone years of baseball training. Thus, the results should be cautiously extrapolated to non-well-trained athletes. Second, PC was a novel treatment to the players; hence, the results may not be appropriate for extrapolation to populations who are accustomed to cold pain. Therefore, future studies are warranted to determine whether PC would provide an ergogenic effect for cold adaptation in participants performing baseball throwing.

## Conclusions

In conclusion, intermittent PC reduces perceived exertion during baseball throwing regardless of whether trained baseball players experience cooling-induced light-to-moderate pain. Furthermore, for trained baseball players who do not feel pain during intermittent PC treatment, inter-set PC (10°C, 2.5 min) elicits higher arousal levels, greater mean throwing velocity, higher maximal throwing velocity, and better throwing accuracy during repeated baseball throwing. However, intermittent PC does not provide an ergogenic effect in well-trained baseball players who experience cooling-induced pain during repeated baseball throwing. Additionally, athletes exhibit decreased mean throwing velocity and maximal throwing velocity. The findings of this study show that a pain-free baseball player from PC could use PC as an ergogenic aid to enhance baseball throwing performance; the presence or absence of pain after PC can serve as an initial assessment of whether to use PC as an ergogenic aid strategy.
